# Appropriateness of Antibiotic Prescription Among Children Under 5 Years: A Cross‐Sectional Study in a Ghanaian Regional Hospital

**DOI:** 10.1002/hsr2.70761

**Published:** 2025-04-29

**Authors:** Beatrice Obour, Glover Asiedu Appiah, Emmanuel Ayitey Tagoe, Harriet Affran Bonful

**Affiliations:** ^1^ Department of Epidemiology and Disease Control, School of Public Health University of Ghana Accra Ghana; ^2^ Department of Monitoring and Evaluation, School of Public Health University of Ghana Accra Ghana; ^3^ Department of Medical Laboratory Sciences, School of Biomedical and Allied Health Sciences University of Ghana Accra Ghana

**Keywords:** antibiotic resistance, antibiotics, inappropriate antibiotic prescription, infection, outpatient

## Abstract

**Background and Aim:**

Misuse of medications, particularly antibiotics, severely impacts the standard of care and can result in antibiotic resistance. Antibiotic resistance is a growing problem in Ghana, compromising patient outcomes. This study aims to assess antibiotic prescription pattern and level of inappropriateness based on Standard Treatment Guideline (STG) recommendations in children under 5 years.

**Methods:**

An analytical cross‐sectional study design was used to assess antibiotic prescription in children under 5 years attending the Wa Regional Hospital in Ghana. Medical records of pediatric outpatients from January to December 2022 were reviewed. Patients' sociodemographic characteristics, prescribed antibiotics, principal diagnosis, dose, and duration were extracted using a semi‐structured form. Prescriptions with clinical indication, dose, and duration which did not meet the requirements of the STG were coded as inappropriate. Data were analyzed and factors associated with inappropriate antibiotic prescription were determined using logistic regression.

**Results:**

Children's mean age was 2.95 ± 1.20 years, with males comprising 54.5%. Most patients had NHIS coverage (90.6%). This study reports 62.7% (266/424) inappropriate antibiotic prescriptions in children under 5 years with infections. The most common classes of antibiotics wrongly prescribed were cephalosporin 54.3% (230/424), penicillin 21.7% (92/424), and aminoglycoside 12.5% (53/424). The class of principal diagnoses likely to have inappropriate antibiotic prescriptions included respiratory tract infections (aOR = 3.82; 95% CI = 2.13, 6.85; *p* < 0.0001) and urinary tract infections (aOR = 0.21; 95% CI = 0.11, 0.41; *p* < 0.0001).

**Conclusion:**

Prevalence of inappropriate prescription of antibiotics was high among the study population, and this was strongly associated with respiratory and urinary tract infections. This study highlights the need to monitor antibiotic prescriptions in hospitals to ensure treatment effectiveness and combat antimicrobial resistance.

## 1 Introduction

Inappropriate antibiotic prescription, defined as the unnecessary use and inappropriate selection, dosing, and duration of prescribed antibiotics, is a global health concern, particularly among pediatric populations [1]. Globally, around 50% of antibiotic prescriptions are inappropriate. Various studies have shown high rates of inappropriate antibiotic prescription in countries such as the United States, the Netherlands, and England, ranging from 30% to 76%, emphasizing the urgent need for intervention and adherence to professional guidelines [2, 3, 4, 5, 6].

The prevalence of inappropriate antibiotic prescription is exacerbated in Africa due to the high burden of infections and the limited availability and high cost of alternative antibiotics [7]. Studies in countries like Nigeria, Cameroon, and Uganda link this issue to nonadherence to essential medicine lists and national Standard Treatment Guidelines (STG) [8, 9, 10]. In Ghana, over half of the medications, including antibiotics, are inappropriately prescribed, administered, or sold [11, 12, 13]. This misuse is notably high in public health facilities and more pronounced in rural areas [14].

In pediatric populations, several sociodemographic factors such as age, sex, and the educational status of the patient's parent influence antibiotic prescription patterns [15]. Prescribers' patterns are also influenced by their qualifications, experience, methods of updating knowledge, and in‐service training [16]. Health system factors like drug availability, performance‐based financing, patient turnout, and healthcare service cost further impact antibiotic prescription patterns in children under 5 years [9].

Inappropriate antibiotic prescriptions in children under 5 years can lead to severe consequences such as mortality, antimicrobial resistance (AMR), and metabolic disorders [17, 18, 19]. AMR, estimated to cause 10 million deaths annually by 2050, is a significant challenge to effective infection treatment worldwide [20, 21]. In Ghana, AMR threatens antibiotic treatment efficacy and compromises patient outcomes, particularly in rural areas where healthcare infrastructure is limited [22, 23].

Several studies have assessed the appropriateness of antibiotic prescriptions based on standards like the BNF and WHO International Network of Rational Use of Drugs [12, 15, 21, 24, 25]. However, there is a scarcity of studies assessing antibiotic prescription appropriateness based on Ghana's STG, especially in rural areas [26]. The STGs are systematically developed recommendations by the Ministry of Health (MoH) designed to assist healthcare providers in selecting appropriate treatments for specific clinical conditions. The current (7th) edition of the STG is the MoH's officially approved prescribers' and dispensers' guide for levels of healthcare in Ghana. The STG provides revised age and disease‐specific doses and treatment durations to promote uniform and optimal prescribing practices [27]. The STGs typically represent a consensus on the most effective treatment options within a healthcare system and aim to positively influence prescribing behavior across all levels of care [27]. Regular assessment of antibiotic prescription in rural health facilities based on STG recommendations can help identify specific drug use problems, sensitize clinicians on rational medicine use, and provide policymakers with relevant information to review medicine‐related policies in Ghana [28]. Currently, little information exists on the adherence to STG in rural communities in Ghana.

The Upper West Region is one of the most rural regions in Ghana, with only 26.4% of its population living in urban areas [29]. The region's referral hospital, located in Wa, provides general and specialized healthcare services to a population of about 901,502. The region has experienced a high under‐5 years mortality rate (40 deaths/1000 live births) for nearly a decade. Infections leading to fever, diarrhea, and symptoms of acute respiratory infection are significant contributors to under‐5 mortality in rural communities [30]. Increased infections have necessitated a corresponding rise in antibiotic prescriptions, but adherence to guidelines in these burdened rural communities is not well‐documented. This study aims to assess antibiotic prescription patterns and the level of inappropriateness based on STG recommendations in children under 5 years at the Wa Regional Hospital. The findings will help inform strategies to improve antibiotic prescribing practices and contribute to better health outcomes for children under 5 years in rural Ghana.

## 2 Materials and Methods

### 2.1 Study Design, Study Setting, and Study Period

This study was an analytical retrograde cross‐sectional study involving a sample of 424 medical records of children under 5 years old from January to December 2022 at Wa Regional Hospital in Ghana. All clinical information (principal diagnosis, daily dose, and duration) and sociodemographic (age, sex, weight, temperature, and NHIS status) features of children were extracted from the sampled folders. Only outpatient folders for children under 5 years old were sampled for more representative common infections. Medical records containing prescriptions of topical preparation, eye drop, and ear‐drop antibiotics were excluded due to their minimal systemic effects.

### 2.2 Sample Size Determination

The total number of outpatient encountered at the pediatric department from January to December 2022 were 4808 according to the Health Information Unit (HIU) at Wa Regional Hospital. Using Cochran's formular and assuming the percentage of outpatients prescribed antibiotics in the pediatric department in 2022 was 50%, a sample of 424 patients with at least one prescribed antibiotic will give us the width of a 95% confidence interval (95% CI) of 9%–10% if the prevalence of unnecessary antibiotics prescription is in the range of 30%–70% [31].

### 2.3 Sampling

The study employed systematic random sampling to select 424 folders of children with at least one prescribed antibiotic in the time period of interest. A total of 4808 folders were obtained from the HIU at Wa Regional Hospital, and sequentially numbered. The first folder was randomly selected from the first five sequential numbers, followed by the selection of every 5th folder thereafter until the desired sample size was achieved.

### 2.4 Classification of Appropriateness

The primary dependent variable in this study was the appropriateness of prescribed antibiotics, evaluated across three domains: clinical indication (therapeutic use), daily dose, and duration of therapy. Each domain was assigned a score based on recommendations from the STG. Prescribed antibiotics adhering to STG recommendations were coded as 1, while those deviating were coded as 0 within each domain. Antibiotic prescriptions were categorized based on their cumulative score: those scoring 3 were deemed appropriate, while scores of 0, 1, or 2 were considered inappropriate.

### 2.5 Data Collection

Data were obtained from three sources: folders, prescribers, and the health facility. First, information from the patient folder was sought from the Health Information Department at the Wa regional hospital. Data were extracted by a team of trained pharmacists. Information regarding the patient characteristics (age, sex, weight, and temperature) and clinical data (diagnosed disease, antibiotics prescribed, route of administration, dose, and duration of prescribed antibiotics) were extracted. The data extraction was performed using the Kobo Collect Application version v2021. 2.4. Kobo Collect is an open‐source Android app for extracting and collecting survey data.

Subsequently, a semi*‐*structured questionnaire was used to collect data on prescriber factors (qualification, years of experience, in‐service training, method and sources of updating knowledge, and influence from medical representatives) from prescribers in the form of a face‐to‐face interview. Informed consent was obtained from prescribers who provided care to the outpatient at the Pediatric Department of the Wa Regional hospital.

Finally, the presence of STG was determined by the availability of the printed copy of the STG in the consulting rooms at the pediatric outpatient department (OPD).

### 2.6 Data Processing and Analysis

Data were processed daily in Microsoft Excel (2016 Microsoft Corporation) and STATA version 15 was used for analysis. A descriptive analysis was used to quantify the level of inappropriate antibiotic prescription in children under 5 years. A *χ* ^2^ analysis was used to determine the association between factors assessed and inappropriate antibiotic prescription while logistic regression was used to measure the strength of association between inappropriate antibiotic prescribing and associated factors. Adjusted odds ratio (AOR) and 95% CI were used to present the results significance.

### 2.7 Ethical Considerations

Ethical clearance was sought from Ghana Health Service Ethics Review Committee, Research and Development Division, Accra. Also, administrative permission was sought from the management of the Wa Regional Hospital prior to the study. Written informed consent was obtained from the prescribers, and patients' confidentiality was diligently maintained throughout the data collection process.

## 3 Results

### 3.1 Sociodemographic Characteristics of Patients

Overall, a total of 424 patient folders of children under 5 years were analyzed. The mean age of the patients was 2.95 ± 1.20 years. Male patients (54.5%) were more compared with female patients (45.5%). The mean weight and temperature of patients were 12.35 ± 4.58 kg and 37.44°C ± 14.93°C, respectively. A high proportion of patients (90.6%) had NHIS insurance. The summary of patient sociodemographic characteristics is provided in Table 1. The most common principal diagnosis of patients studied were urinary tract infections (UTIs) (21.5%), otitis media (16.8%), gastroenteritis (15.3%), pneumonia (13.7%), common cold (7.1%), sepsis (6.6%), and gastritis (3.5%) making respiratory tract infections (RTIs) (44.1%) the most common class of principal diagnosis. The principal diagnosis of children under 5 years at Wa Regional Hospital are summarized in Table 2.

**Table 1:** Sociodemographic characteristics of children under 5 years with at least one antibiotic prescription at Wa Regional Hospital in 2022.

Characteristics	Frequency (%) (*N* = 424)
Sex of patients	
Males	193 (45.5)
Females	231 (54.5)
Age category (years)	
Less than 2	79 (18.6)
2–3	203 (47.9)
4–5	142 (33.5)
National Health Insurance Scheme	
Yes	384 (90.6)
No	40 (9.4)

	Mean ± SD (*N* = 414)
Age (years)	2.95 ± 1.20
Body weight (Kg)	12.35 ± 4.58
Body temperature (°C)	37.44 ± 14.93

**Table 2:** Principal diagnosis of children under 5 years with at least one antibiotic prescription at the Wa Regional Hospital in 2022.

Classification of principal diagnosis	Frequencies (*N* = 424)	Percentages (%)
Gastrointestinal tract infection	81	19.10
Others	35	8.25
Respiratory tract infection	187	44.10
Skin infection	29	6.84
Urinary tract infection	92	21.70

Principal diagnosis	Frequencies (*N* = 424)	Percentages (%)
Ameobic dysentery	1	0.2
Asthma	2	0.5
Boils	5	1.2
Bronchitis	4	0.9
Burns	2	0.5
Cellulitis	7	1.7
Common cold	30	7.1
Dermatitis	5	1.2
Gastritis	15	3.5
Gastroenteritis	65	15.3
Helmenthiasis	1	0.2
Impetigo	5	1.2
Insect bite	1	0.2
Malaria	5	1.2
Neonatal jaundice	1	0.2
Otitis media	71	16.8
Pharyngitis	4	0.9
Pneumonia	58	13.7
Scabies	2	0.5
Sepsis	28	6.6
Sickle cell anemia	1	0.2
Syphilis	1	0.2
Tinea pedis	1	0.2
Tonsilitis	5	1.2
URTI	13	3.1
UTI	91	21.5

Abbreviations: UTI, urinary tract infection; URTI, upper respiratory tract infection.

### 3.2 Antibiotics Prescribed

**Figure 1:**
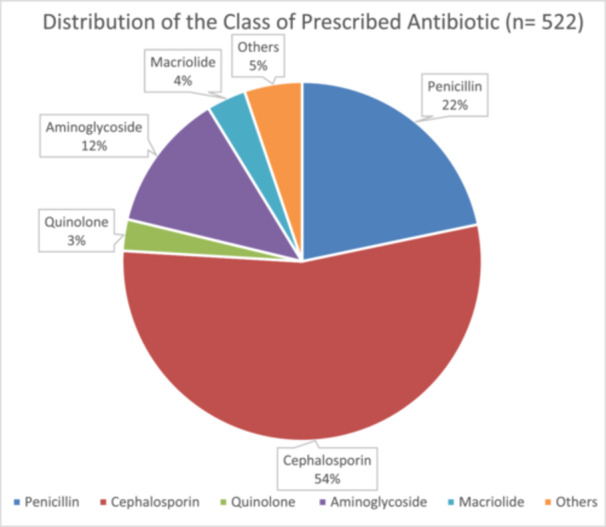
Distribution of the class of prescribed antibiotic.

**Figure 2:**
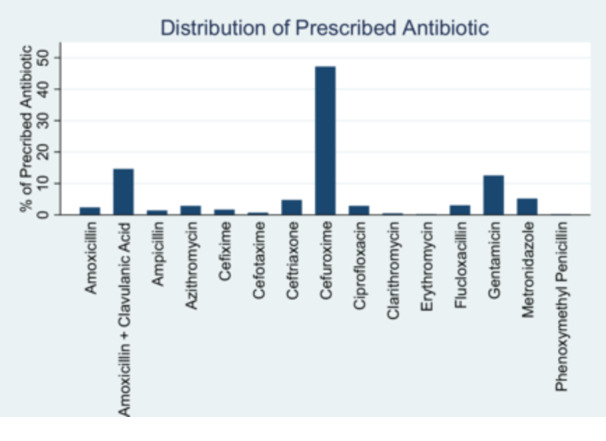
Distribution of patients by prescribed antibiotics (*n* = 522).

The majority of 424 children under 5 years were prescribed just one antibiotic (77.1%), followed by 22.4% of patients with two antibiotics, and only 0.5% of patients were prescribed three antibiotics within a visit. Among the total 522 prescriptions of antibiotics, the top 3 classes from which antibiotics were prescribed were cephalosporin (54.3%, *n* = 230), penicillin (21.7%, *n* = 92), and aminoglycoside (12.5%, *n* = 53) (Figure 1). Cefuroxime (47.2%, *n* = 200), amoxicillin + clavulanic acid (14.5%, *n* = 62), gentamicin (12.5%, *n* = 53), metronidazole (5.2%, *n* = 22), and ceftriaxone (4.7%, *n* = 20) were the topmost 5 specific types of antibiotics prescribed (Figure 2). The distribution of prescribed antibiotic is summarized in Table 3. The proportion of prescribed antibiotics with the oral route of administration (80.9%) was higher than the proportion of prescribed antibiotics with the parenteral route of administration (19.1%).

**Table 3:** Distribution of prescribed antibiotics to children under 5 years at Wa Regional Hospital in 2022.

Class of prescribed antibiotics	Antibiotic prescribed	Frequencies (*N* = 522)	(%) of antibiotic type	(%) of antibiotic class
Penicillin	Amoxicillin	10	2.4	21.71
	Amoxicillin + clavulanic acid	62	14.6	
	Ampicillin	6	1.4	
	Flucloxacillin	13	3.1	
	Phenoxymethylpenicillin	1	0.2	
Cephalosporin	Cefixime	7	1.7	54.25
	Cefotaxime	3	0.7	
	Ceftriaxone	20	4.7	
	Cefuroxime	200	47.2	
Quinolone	Ciprofloxacin	12	2.8	2.83
Aminoglycoside	Gentamicin	53	12.5	12.5
Macrolide	Azithromycin	12	2.8	3.54
	Clarithromycin	2	0.5	
	Erythromycin	1	0.2	
Others	Metronidazole	22	5.2	5.19

### 3.3 Characteristics of Prescribers

At the time of the study, a census of three pediatric outpatient prescribers were interviewed (two general doctors and one pediatrician). The mean years of experience of the prescribers was 6.33 ± 3.51 years. Two out of the three prescribers received in‐service training on antibiotic prescribing. All three prescribers update their knowledge with British National Formula (BNF) and Medscape while two of the prescribers update their knowledge with the STG or other means such as articles from the internet. Characteristics of prescribers are summarized in Table 4. All three prescribers acknowledged occasionally prescribing antibiotics to children under 5 years based on laboratory findings. Additionally, prescribers strongly disagreed with the influence of medical representative on their prescribing patterns for antibiotics.

**Table 4:** Baseline characteristics of prescribers.

Characteristics	Frequencies (*N* = 3)	Percentages (%)
Qualification of prescriber		
General doctors	2	66.7
Pediatricians	1	33.3
In‐service training on antibiotic prescribing		
Yes	2	66.7
No	1	33.3
Source or method of updating knowledge		
STG	2	66.7
BNF	3	100.00
Medscape	3	100.00
Others	2	66.7

Abbreviations: BNF, British National Formular; STG, Standard Treatment Guideline.

### 3.4 Availability of STGs in Consulting Rooms

The study found that the two available consulting rooms in the pediatric OPD did not have a hard copy of the STG.

### 3.5 Appropriateness of Prescribed Antibiotics

**Figure 3:**
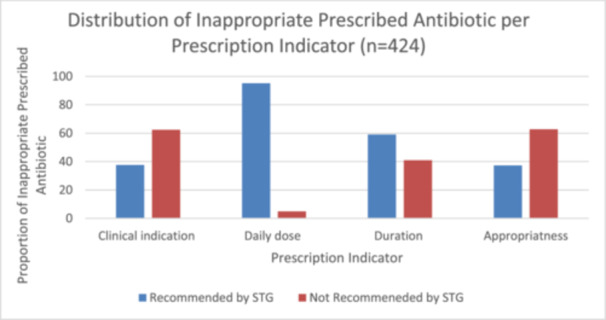
Distribution of inappropriate prescribed antibiotics per prescription indicators.

Categorization of prescribed antibiotics as appropriate or inappropriate based on the three prescription indicators (clinical indications, daily dose, and duration) as recommended by the STG is provided in Table 5. The proportion of prescribed antibiotics which was not recommended under STG based on clinical indication (39.4%) was higher than the respective proportions for duration (25.9%) and daily dose (3.1%). The proportion of prescribed antibiotics which were inappropriate (62.7%) based on the three prescription indicators which was not recommended under STG were higher than those which were appropriate (37.3%). The proportion of inappropriate prescribed antibiotics which was not recommended under STG based on clinical indications (62.4%) (95% CI 57.9, 67.4) was higher than the respective proportions for duration (59.0%) and daily dose (4.9%) (Figure 3).

**Table 5:** Categorization of prescribed antibiotics into appropriate and inappropriate based on three prescription indicators that meet the STG recommendation.

Characteristics	Frequencies (*N* = 424)	Percentages (%)
Clinical indication of prescribed antibiotics		
Recommended by STG	257	60.6
Not recommended by STG	167	39.4
Daily dose of prescribed antibiotics		
Recommended by STG	411	96.9
Not recommended by STG	13	3.1
Duration of prescribed antibiotics		
Recommended by STG	314	74.1
Not recommended by STG	110	25.9
Appropriateness of prescribed antibiotic		
Appropriate	158	37.3
Inappropriate	266	62.7

Abbreviation: STG, Standard Treatment Guideline.

### 3.6 Association Between Appropriateness of Prescribed Antibiotic and Other Variables

Table 6 showed that there was no statistically significant relationship between the appropriateness of prescribed antibiotics and the sex, age category and NHIS status of the patient. The class of principal diagnosis of children under 5 years were significantly associated with the appropriateness of prescribed antibiotics (*p* < 0.0001) (Table 7). Table 8 reveals the association between inappropriate prescribed antibiotics by sex, age category, NHIS status, weight, temperature and class of principal diagnosis using univariable and multivariable logistic regression. There was no statistically significant relationship between inappropriate prescribed antibiotics and sex, age category, weight, temperature and NHIS status. The odds of inappropriate prescribed antibiotics were increased 3.82 times in patients diagnosed with RTIs compared to those diagnosed with gastrointestinal tract infection after adjusting for other variables (aOR = 3.82; 95% CI = 2.13, 6.85; *p* < 0.0001). Similarly, patients with UTIs had 79% reduced odds of being prescribed inappropriate antibiotics in contrast to patients with gastrointestinal tract infection after adjusting for other covariates (aOR = 0.21; 95% CI = 0.11, 0.41; *p* < 0.0001).

**Table 6:** Comparison of appropriateness of prescribed antibiotics with inappropriateness based on sociodemographic characteristics.

Characteristics	Appropriate *N* (%)	Inappropriate *N* (%)	*p*
Sex of patient			
Female	76 (39.4)	117 (60.6)	0.411
Male	82 (35.5)	149 (64.5)	
Age category			
< 2	22 (27.9)	57 (72.2)	0.143
2–3	78 (38.4)	125 (61.6)	
4–5	58 (40.9)	84 (59.2)	
NHIS status			
Insured	146 (38.0)	238 (62.0)	0.318
Noninsured	12 (30.0)	28 (70.0)	

**Table 7:** Association between appropriateness of prescribed antibiotics and class of principal diagnosis.

Class of principal diagnosis	Appropriate *n* (%)	Inappropriate *n* (%)	*p*
Gastrointestinal tract infection	37 (45.68)	44 (54.32)	0.000
Respiratory tract infection	34 (18.18)	153 (81.82)	
Skin infection	14 (48.28)	15 (51.72)	
Urinary tract infection	73 (79.35)	19 (20.65)	
Others	0 (0.0)	35 (100)	
Principal diagnosis			
Gastritis	1 (100)	0 (0.0)	0.000
Gastroenteritis	0 (0.0)	2 (100)	
Helminthiasis	4 (80)	1 (20)	
Impetigo	0 (0.0)	4 (100)	
Insect bite	0 (0.0)	2 (100)	
Malaria	5 (71.4)	2 (28.6)	
Neonatal jaundice	0 (0.0)	30 (100)	
Otitis media	0 (0.0)	5 (100)	
Pharyngitis	7 (46.7)	8 (53.3)	
Pneumonia	29 (44.6)	36 (55.4)	
Scabies	0 (0.0)	1 (100)	
Sepsis	5 (100)	0 (0.0)	
Sickle cell anemia	0 (0.0)	1 (100)	
Syphilis	0 (0.0)	5 (100)	
Tinea pedis	0 (0.0)	1 (100)	
Tonsilitis	15 (21.2)	56 (78.9)	
URTI	1 (25)	3 (75)	
UTI	17 (29.3)	41 (70.7)	

**Table 8:** Univariable and multivariable logistic regression for inappropriate prescribed antibiotics.

Characteristics	Inappropriate prescribed antibiotics			
	OR (95% CI)	*p*	aOR (95% CI)	*p*
Sex of patient				
Female	1 (base)		1 (base)	
Male	1.18 (0.80–1.75)	0.411	0.80 (0.49–1.30)	0.369
Age category				
< 2	1 (base)		1 (base)	
2–3	0. 62 (0.35–1.09)	0.097	0.86 (0.45–1.74)	0.672
4–5	0.56 (0.31–1.01)	0.055	0.76 (0.36–1.61)	0.476
NHIS status				
Insured	1 (base)		1 (base)	
Noninsured	1.43 (0.71–2.90)	0.320	1.06 (0.44–2.58)	0.890
Class of principal diagnosis				
Gastrointestinal tract infection	1 (base)		1 (base)	
Respiratory tract infection	3.78 (2.13–6.72)	0.000	3.82 (2.13–6.85)	0.000
Skin infection	0.90 (0.39–2.11)	0.810	0.81 (0.34–1.93)	0.631
Urinary tract infection	0.22 (0.11–0.43)	0.000	0.21 (0.11–0.41)	0.000*
Others	1	—	1	—
Weight of patient	0.97 (0.93–1.01)	0.183	0.99 (0.98–1.010)	0.407
Temperature of patient	1.01 (0.97–1.05	0.581	1.01 (0.99–1.02)	0.349

*Note:* * indicate *p* < 0.05 is considered statistically significant.

Abbreviations: aOR, adjusted odds ratio; CI, confidence interval; OR, odds ratio.

## 4 Discussion

Our study findings revealed a high proportion (62.7%) of prescribed antibiotics were inappropriate based on recommendations under the STG in children under 5 years. The clinical indication, daily dose, and duration of prescribed antibiotic have an influence on the appropriateness of prescribed antibiotics [32]. Prescription of antibiotics should, therefore, be guided by these prescription indicators. A cross‐sectional study on antibiotic prescription in the Tamale Teaching Hospital in Ghana revealed that the most common prescription error found was the duration of treatments (29.6%) [25]. However, this study revealed that the proportion of prescribed antibiotics which was not recommended under STG based on clinical indication (39.4%) was higher than the respective proportions for duration (25.9%) and daily dose (3.1%). This contrast may be due to the fact that the aforementioned study used the BNF as a reference for appropriateness and also conducted the study in other departments within the hospital.

Similar to our findings, a cross‐sectional study conducted in 2013 for children under the age of 14 years in a Spanish hospital's emergency department showed the proportion of inappropriate antibiotic prescriptions was 51.9%, wrong antibiotic choice was 35.2%, and inaccurate posology was 24.1% [33]. Other studies also corroborate with our findings which suggests that the total inappropriate antibiotic prescribed may approach 50% of all outpatient antibiotic use [34]. The 3.1% inappropriate dosing is approximately equal to the 3.3% for inappropriate dosing frequency but the 25.9% of inappropriate duration was higher than the 19.0% for inappropriate treatment duration in an outpatient evaluation of prescribed antibiotic at the Tema Polyclinic [26]. This observation may be explained by the perception of pediatricians that healthcare pressure is an influential factor in the prescribing behavior, forcing them to prescribe antibiotics for a rapid cure in unjustified circumstances. The misuse of antibiotics by pediatricians has been associated with the lack of education on antibiotic prescription and the limited use of clinical guidelines. Not prescribing an antibiotic in the presence of a potentially severe disease generated more fear than an unnecessary prescription [16].

The top 3 classes from which antibiotics were prescribed were cephalosporin, penicillin, and aminoglycoside. Amoxicillin, amoxicillin–clavulanic acid, cefuroxime, ceftriaxone, and gentamicin are antibiotics that have been identified as the most prescribed antibiotics in four studies conducted in various locations in Ghana [12, 15, 24, 26]. Cefuroxime, amoxicillin + clavulanic acid, gentamicin, metronidazole, and ceftriaxone were the topmost 5 specific types of antibiotics prescribed in this study.

One antibiotic was mostly prescribed to patients per visit which is within the ideal WHO standard of < 2 antibiotics/visit [35]. The proportion of prescribed antibiotics with the oral route of administration (80.9%) was higher than the proportion of prescribed antibiotics with the parenteral route of administration (19.1%). This observation, given the risks connected with injectable use, such as necrosis, anaphylactic shock, and others, is a safe alternative [36]. Also, orally taken medications are also more convenient and easier to administer without the involvement of a trained personnel.

The average age and weight of children studied was 2.9 ± 1.2 years and 12.35 ± 4.58 kg, respectively. A study in the Kintampo Municipal Hospital in Brong Ahafo region of Ghana revealed that antibiotics are commonly prescribed in children under 5 years [14]. This observation can be explained by their underdeveloped immune system and vulnerability to various communicable and contagious diseases. The average temperature of children under 5 years prescribed antibiotics was 37.43 ± 14.93°C indicating the absence of raised temperature. Patient temperature was found as one of the influential factors of antibiotic prescribing in the Danish general practice [37]. Our findings noted male patients received more prescribed antibiotic compared to female patients. This finding was similar to the study by Nasso and colleagues which identified a higher proportion of male patients on prescribed antibiotics [38]. Most of the patients were NHIS insured (90.6%). This is consistent with the 90.3% obtained in a related study in Ghana's Eastern area [12]. One of the primary goals of a National Health Insurance Scheme (NHIS) is to ensure access to healthcare services by removing the financial barrier [39].

UTI, otitis media, gastroenteritis, pneumonia, and common cold were the top 5 conditions treated with antibiotics in children under 5 years. The aforementioned diagnoses are similar to those observed in similar research studies conducted in various parts of Ghana, for which antibiotics were prescribed [12, 14, 24, 26].

A study conducted at Kintampo Hospital in the Brong Ahafo region of Ghana found that antibiotics were inappropriately prescribed for RTIs such as the common cold, pneumonia, and otitis media [14]. In this study, antibiotics were inappropriately prescribed for otitis media, pharyngitis, pneumonia, and unspecified URTI. The RTIs aforementioned for which prescription indicators do not meet the STG recommendation were highest for duration compared to clinical indication and daily dose with the exception of unspecified URTI. The findings of this study underscore the need to sensitize prescribers on appropriate prescribing of antibiotics especially the duration.

Several studies on antibiotic use among patients with UTI revealed a significant level of inappropriate prescribing based on nonrecommended dose and duration of the prescribed antibiotics [40, 41, 42]. In this study, nonrecommended clinical indication and duration were the most common prescription errors associated with inappropriate antibiotic prescribing in patients with UTI.

Pediatricians, as the clinicians most intensively trained and experienced in child health, are the natural leaders of pediatric primary healthcare. In this study only one pediatrician was available at the pediatric OPD. Their specialized education, training, and skill in child health make them essential in all aspects of children care, including pediatric antibiotic prescribing [43]. The mean years of experience of the prescribers was 6.33 ± 3.51 years. Results from the regressions of a study showed a consistent association between pediatrician's antibiotic prescribing decisions and experience: more experienced physicians chose shorter therapies and deviated less from the experts' recommendation [44]. Prescribers received in‐service training on antibiotic prescribing (66.7%), used reference materials in updating their knowledge, sometimes prescribed antibiotics based on laboratory investigation and strongly disagreed to the influence of medical representative on their prescribing patterns for antibiotics. These factors were associated to inappropriate antibiotic prescribing in a similar study [16].

The limited use of clinical guidelines may be worsened by the absence of STGs in the consulting rooms. A study in Kenya in 2017 identified that only 33.9% of antibiotic encounters were appropriately prescribed and identified nonavailability of guidelines and/or poor compliance as the reasons [45]. In this study, 37.3% of prescribed antibiotics were appropriate and hard copies of the STG were not available in either of the two pediatric OPD consulting rooms. These observations call for the provision of new STGs in the consulting rooms (as well as personal copies for prescribers) and training or a refresher training for prescribers to conform to the STG in prescribing antibiotics for patients as appropriate use of antibiotics is needed to curb the rising problem of AMR.

## 5 Strength and Limitation of Study

The study was conducted in a regional hospital which serves as a referral hospital for the inhabitants of the Upper West region hence diverse medical conditions are treated and a fair representation of the region is achieved.

This study was conducted in the northern belt of Ghana hence findings may not apply to middle and southern belt of Ghana. Prescriber factors were not associated with specific prescribing practices. It therefore remains unclear if practices as found apply equally to all prescribers.

Another limitation is the potential for information and misclassification bias because the study was based on one regional hospital. Other key indicators such as laboratory and clinical assessment were not used in determining the validity or otherwise of the diagnosis made.

## 6 Conclusion

This study concludes that the prevalence of inappropriate prescribed antibiotics was high for children under 5 years and wrong prescription was strongly associated with RTIs and UTIs. This study highlights the need to monitor antibiotic prescriptions in hospitals to ensure treatment effectiveness and combat AMR.

## Author Contributions

**Beatrice Obour:** conceptualization, investigation, writing – original draft, formal analysis, resources. **Glover Asiedu Appiah:** writing – original draft, data curation. **Emmanuel Ayitey Tagoe:** formal analysis, writing – review and editing. **Harriet Affran Bonful:** conceptualization, formal analysis, writing – review and editing, supervision.

## Ethics Statement

Ethical clearance for the study was obtained from the Ghana Health Service Ethics Review Committee (Approval Number: GHS‐ERC: 038/08/23), and administrative permission was obtained from the Management of Wa Regional Hospital.

## Conflicts of Interest

The authors declare no conflicts of interest.

## Transparency Statement

The lead author Harriet Affran Bonful affirms that this manuscript is an honest, accurate, and transparent account of the study being reported; that no important aspects of the study have been omitted; and that any discrepancies from the study as planned (and, if relevant, registered) have been explained.

## Data Availability

The data will be made available upon reasonable request from the corresponding author (habonful@ug.edu.gh).
